# Supplementary figures and images for: Chronic peptide-based GIP receptor inhibition exhibits modest glucose metabolic changes in mice when administered either alone or combined with GLP-1 agonism
Source: PLoS One. 2021 Mar 31;16(3):e0249239. doi: 10.1371/journal.pone.0249239 (PMC8011784; doi:10.1371/journal.pone.0249239)

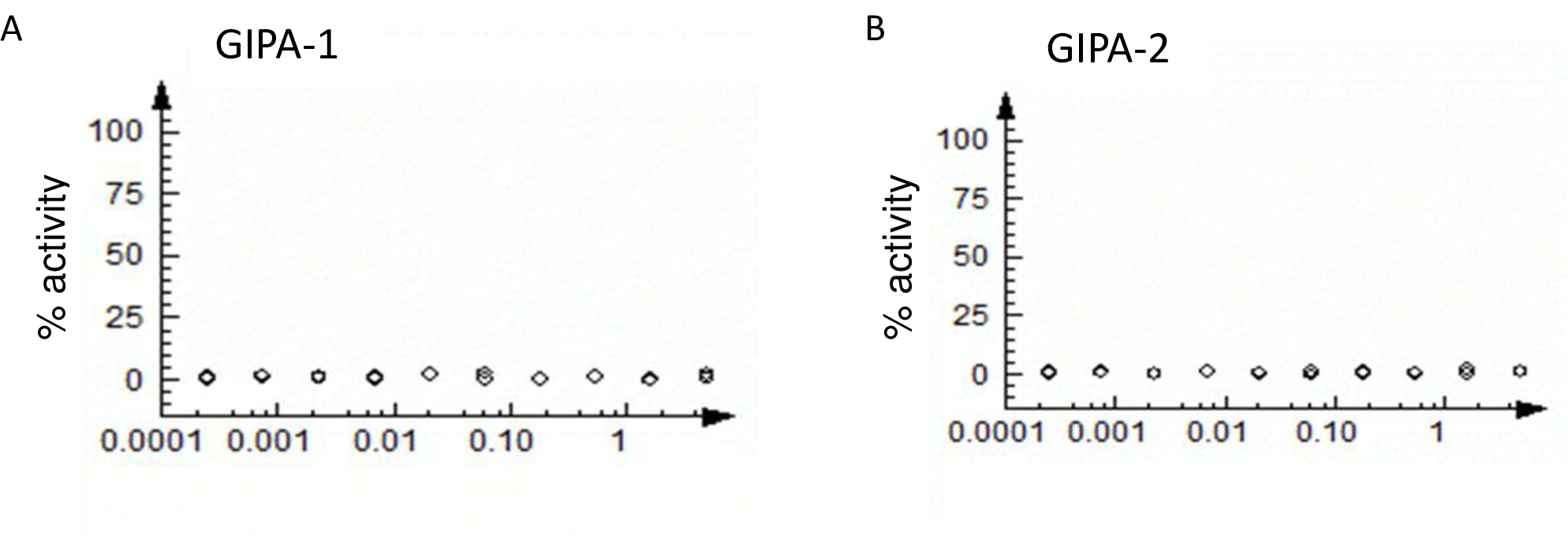

Supplement: S1 Fig — Representative concentration response curves for stimulation of cAMP accumulation by GIPA-1 (A) and GIPA-2 (B) in CHO-K1 cells expressing mouse GIPR, showing that neither is acting as partial agonist. (TIF) [file pone.0249239.s001.tif]

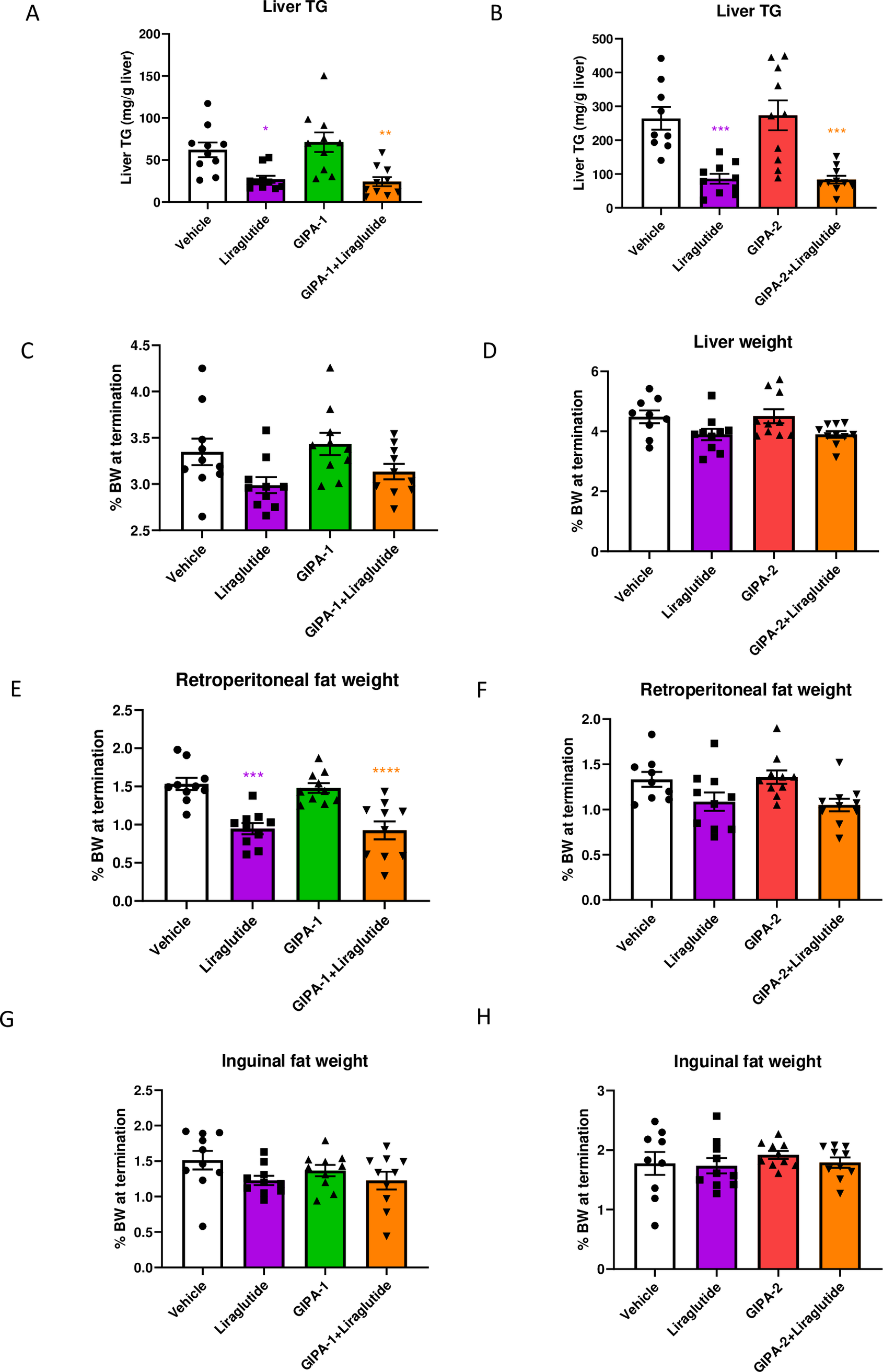

Supplement: S2 Fig — Effects of GIPA-1 and GIPA-2 on liver TG (A and B), liver weight (C and D), retroperitoneal fat weight (E and F) and inguinal fat weight (G and H) following administration of vehicle, liraglutide, GIPR antagonist, and liraglutide in combination with the GIPR antagonist for 28 days in DIO mice. Group sizes are n = 10, and data are represented as mean ± SEM. Statistical analysis was calculated using one-way ANOVA with Dunnett’s post-hoc test. *: p< 0.05, **: p < 0.01, ***: p < 0.001, ****: p<0.0001 compared to vehicle. (TIF) [file pone.0249239.s002.tif]
