# Supplementary material for: Chronic peptide-based GIP receptor inhibition exhibits modest glucose metabolic changes in mice when administered either alone or combined with GLP-1 agonism
Source: PLoS One. 2021 Mar 31;16(3):e0249239. doi: 10.1371/journal.pone.0249239 (PMC8011784; doi:10.1371/journal.pone.0249239)
Supplement: Attachment — Submitted filename: 2021-03-12 Response to reviewers.docx [file pone.0249239.s004.docx]

**Response to reviewers: PONE-D-20-36396**

**Journal Requirements**:

**Please review your reference list to ensure that it is complete and correct. If you have cited papers that have been retracted, please include the rationale for doing so in the manuscript text, or remove these references and replace them with relevant current references. Any changes to the reference list should be mentioned in the rebuttal letter that accompanies your revised manuscript. If you need to cite a retracted article, indicate the article’s retracted status in the References list and also include a citation and full reference for the retraction notice.**

Our reference list has been reviewed and we can confirm that no cited paper has been retracted. Only for reference 28, there was a publisher correction involving addition of one of the author’s name omitted from the authors list in the original published article.

**Reviewers' comments:**

**Reviewer 2**

Thank you very much for taking the time to review our manuscript. We welcome your comments and have addressed the points you raised below and where appropriate updated the manuscript.

**1. Inappropriate protocol for OGTT and ITT in the mice treated chronically with GIPA-1 or GAPA-2 (Fig. 3)**

In your comments for GIPA-2 administration you stated, “If the single injection of GIGA-2 per day is sufficient for inhibiting GIP signaling up to the next 24 hours, the authors might have not needed to treat another shot on the day of OGTT or ITT”. We would like to clarify that on the days of OGTT and ITT animals received only one shot of GIPA-2 (their daily dose, always administrated at the same time as part of their chronic daily dosing).

We understand your concern and we agree that our OGTT and ITT results might influenced by both ‘chronic’ and ‘acute’ effects of GIPA-2 administration under our protocol. To this extend, we have now added a relevant statement in the discussion (5^th^ paragraph, **in bold and blue**). The text now reads:

“Importantly, despite having a potentiating effect on glucose excursion during an OGTT when given alone, GIPA-2 did not inhibit liraglutide’s beneficial glucose-lowering effect when administered in combination, mirroring our GIPA-1 data. **It should be noted that due to the nature of GIPA-2 administration (daily injections), OGTT results might be influenced by both the ‘chronic’ and ‘acute’ effects of GIPA-2.”**

**2. Lack of data on the mechanism of the alteration in HOMA-IR, plasma TG, plasma FFA, and epididymal fat weight by GIPA-1 (Fig.2J and Fig.4)**

As we stated previously, we agree it is important to investigate the molecular mechanisms behind the effects of antagonists in combination with liraglutide in plasma TG, plasma FFA, and the weight of epididymal fat depots to gain a better insight on the role of GIP on fat metabolism. However, we stand behind our previous statement that clarifying these phenotypes properly would require a significant effort and is beyond the scope of the current manuscript. This is now (after your suggestion) also reflected in the manuscript title, stating GIP receptor inhibition exhibits modest effect on glucose metabolism, making it the focus of our study.

**3. Ambiguous conclusion in the title**

Thank you for your suggestion. We have now added “glucose” in the title. The title now reads: “Chronic peptide-based GIP receptor inhibition exhibits modest glucose metabolic changes in mice when administered either alone or combined with GLP-1 agonism”.
